# Mammalian nuclear speckles exhibit stable association with chromatin: a biochemical study

**DOI:** 10.1080/19491034.2021.2024948

**Published:** 2022-02-27

**Authors:** Komal Raina, Basuthkar J Rao

**Affiliations:** a Department of Biological Sciences, Tata Institute of Fundamental Research, Mumbai, India; b Department of Biology, Indian Institute of Science Education and Research (Iiser) Tirupati, Transit Campus: Sree Rama Engineering College, Tirupati, India; c Department of Animal Biology, University of Hyderabad, Hyderabad, India

**Keywords:** Nuclear speckles, chromatin-association, SR proteins, DNase 1, speckle-associated chromatin

## Abstract

Nuclear Speckles (NS) are phase-separated condensates of protein and RNA whose components dynamically coordinate RNA transcription, splicing, transport and DNA repair. NS, probed largely by imaging studies, remained historically well known as Interchromatin Granule Clusters, and biochemical properties, especially their association with Chromatin have been largely unexplored. In this study, we tested whether NS exhibit any stable association with chromatin and show that limited DNAse-1 nicking of chromatin leads to the collapse of NS into isotropic distribution or aggregates of constituent proteins without affecting other nuclear structures. Further biochemical probing revealed that NS proteins were tightly associated with chromatin, extractable only by high-salt treatment just like histone proteins. NS were also co-released with solubilised mono-dinucleosomal chromatin fraction following the MNase digestion of chromatin. We propose a model that NS-chromatin constitutes a “putative stable association” whose coupling might be subject to the combined regulation from both chromatin and NS changes.

**Abbreviations:** NS: Nuclear speckles; DSB: double strand breaks; PTM: posttranslational modifications; DDR: DNA damage repair; RBP-RNA binding proteins; TAD: topologically associated domains; LCR: low complexity regions; IDR: intrinsically disordered regions.

## Introduction

#Current address: Department of Animal Biology, School of Life Sciences, University of Hyderabad, Hyderabad, India

Nuclear Speckles (NS), also known as splicing speckles or inter-chromatin granule

Clusters are one of the many phase-separated condensates present within the nucleus of eukaryotic cells. In this context, phase-separated condensates refer to the membrane-less organelles or aggregates of macromolecules (proteins or RNAs) that exhibit liquid droplet like behavior. These condensates are dynamic and the assembly/disassembly processes are highly regulated. The low-complexity regions (LCRs) or intrinsically disordered regions (IDRs) of certain RNA binding proteins promote multivalent weak interactions, that lead to aggregation/separation of such proteins into different ‘phases’ [1,2]. These particles typically appear as ~20–50 granules of varying sizes, generally spherical, ranging up to several nanometers in diameter in most mammalian cells [3]. These highly dynamic condensates are enriched in mRNA splicing factors, mRNA export proteins, and transcriptional regulators [4], noncoding RNAs such as MALAT1 [5,6] and various other regulatory proteins as well as DNA repair factors, etc. [7,8]. Proteins necessary for the formation of NS have recently been described and include RNA binding proteins SON and SRRM2 [9,10]. The dynamic proteome of NS and the associated PTMs are still not well understood. It is also surmised that the constituent proteins shuttle dynamically from the NS to the transcription sites on chromatin and revert again to the NS via nucleoplasm in response to varying cellular signaling. The details of such switches in the context of cellular aberrations and diseases are mostly missing in the field.

The proximity of genomic loci to NS has been shown to facilitate transcription of the cognate gene loci in the case of HSP70 following heat shock [11–14]. The novel experimental paradigm, in this case, revealed that the HSP70 transgene arrays relocate directionally and reversibly toward NS following the heat shock treatment of cells. It has also been observed that inhibition of transcription or splicing lead to the reversible enlargement of NS particles along with the accumulation of constituent proteins thereby suggesting that NS reorganize during cellular stress responses [15–17]. Whether NS undergo similar changes during DNA damage response (DDR) has not been well characterized so far. Moreover, DDR, being a global cellular response, is expected to elicit a DNA-repair centric rewiring of transcriptional modulation via RNA splicing changes following the remodeling of NS. However, strong experimental support in favor of such a model is still not available. We surmise that NS remodeling during DDR is associated with specific RNA splicing changes possibly involving the dynamic association of NS with nuclear chromatin, a model that has not been well tested so far. This posits a direct query on whether NS exhibit stable anchorage on cellular chromatin at all, which is the main focus of the current study. Historically, NS (interchromatin particles) were thought to be assembled in ‘chromatin-free’ regions of the nucleus and were detected by electron microscopic analysis as ‘clouds of RNA’ [17,18]. In subsequent studies, these interchromatin particles were identified to contain various splicing factors and snRNPs [19–21]. In addition to NS being potential sites for pre-mRNA splicing, recent studies have suggested more active functions of NS ranging from transcription regulation to HIV integration to genome organization [14,22,23]. Over the past few decades, the proximity of NS with active chromatin has been commented upon and a few recent studies also highlight the association of certain genomic loci with NS [3,11–14,24,25].

This study is aimed at addressing whether NS exhibit any properties that are consistent with them being ‘tethered’ to nuclear chromatin or not. We tested this hypothesis using different biochemical approaches: Firstly, we showed that limited and untargeted nicking of the nuclear genome by DNase 1 digestion of chromatin resulted in large-scale morphological changes in NS where all the particles were disrupted into an isotropic distribution of NS proteins in the nucleoplasm, which also result in few nuclei with massively large NS particles due to protein aggregations. We also showed that NS proteins were stably associated with chromatin as evidenced by their release into soluble phase only at high salt treatment, a property reminiscent of tightly chromatin-bound histones. Lastly, our experiment involving MNase release of soluble chromatin also leading to the simultaneous release of NS into the nuclear soluble fraction (without any prior salt treatment) strongly justifies a model where the chromatin anchorage of NS particles constitutes the basis of NS organization and the continuum of ‘NS-chromatin’ forms its constitutive state. We surmise that the dynamic association of NS vis-à-vis specific gene loci becomes an additional layer of regulation that ensues in concert with changes in chromatin as well as NS-proteome. We describe our results and discuss the same in the context of their plausible functional implications.

## Materials and methods

### Cell culture and treatments

Hek293 (CRL-1573, ATCC) Cells were maintained in DMEM (Dulbecco’s Modified Eagle Medium) (11965118, Gibco) supplemented with 10% Fetal Bovine Serum (at 37°C in 5% CO_2._ Cells were treated with Etoposide (10 μM for 4 hours) to induce sublethal DNA damage and with Actinomycin D (5 μg/ml for 4 hours).

### Immunofluorescence and microscopy

Hek293 cells were grown on glass coverslips, treated as per experimental requirements and fixed using 4% Paraformaldehyde for 20 minutes at room temperature, followed by PBS washes. 0.1% TritonX-100 in PBS was used for permeabilization of the fixed samples. Cells were stained with Anti-Sc35 (S4045, Sigma), Tubulin (AC008, Abclonal), γΗ2ΑX (9718, CST), Emerin (ab40688, Abcam), SON (HPA031755, Sigma), and SRRM1 (ab221061, Abcam) antibodies. This was followed by washing the cells in PBS and further incubation with appropriate secondary antibodies at room temperature for 1 hour: donkey anti-rabbit/mouse Alexa 594 or donkey anti-mouse/rabbit Alexa 488 (Molecular Probes, Invitrogen). Following this, coverslips were washed with PBS and mounted on slides using Vectashield Antifade Mounting medium containing DAPI (H–1200–10,Vector Laboratories) which counterstains the nucleus. After mounting, cells were imaged on Leica Sp8 confocal microscope (63X) and processed using FiJi (Image J). IMARIS was used for the number and volumetric quantification of Nuclear speckles based on the immunostaining of NS markers. For statistical analysis, number of nuclei having less than 15 NS (the population showed highest frequency at 15–20 NS/nucleus) in a population of 100 nuclei were quantified for control and various treatment conditions. The significance was tested using T test. For NS volume quantification number of speckles with volume less than 3μm^3^ were quantified and the significance was tested using T test.

### Isolation of nuclei and DNase 1 treatment

Nuclei were isolated using hypotonic lysis of cell membrane followed by sucrose density gradient to purify nuclei as previously described [26]. Briefly, Hek293 cells were grown in 100 mm dishes and treated with Etoposide, Actinomycin D or DMSO. Cells were washed with 1X PBS, trypsinized and centrifuged at 1000 rpm. The pelleted cells were washed with 1X PBS and suspended in hypotonic buffer (0.32 M sucrose, 60 mM KCl,15 mM NaCl, l5 mM MgCl_2,_0.1 mM EGTA,15 mM Tris pH 7.4, 0.1% NP-40) incubated on ice for 15 minutes, layered on a sucrose cushion (1.2 M sucrose, 60 mM KCl,15 mM NaCl,5 mM MgCl_2_, 0.1 mM EGTA, 15 mM Tris pH 7.4) and centrifuged for 20 minutes at 10,000 × g, 4°C. The nuclear pellet was washed with 1X PBS and fixed as previously described or resuspended in DNase 1 buffer (10 mM Tris-HCl pH7.6, 2.5 mM MgCl_2_, 0.5 mM CaCl_2_). The resuspended nuclei were treated with DNase 1(1 U/ul) at 37°C for different durations. The treatment was stopped by adding 20 mM EGTA and nuclei were collected by centrifugation at 400 g. The supernatant was retained and the released DNA was purified using a PCR purification kit and ran on 1.5% agarose gel. The nuclear pellet was washed with PBS and fixed with 4% PFA. Following fixation, the DNase 1 treated nuclei were processed as described earlier.

### PolyA mRNA fluorescence in situ hybridization (FISH)

Cells grown on coverslips or purified nuclei were treated with DNase 1(1 U/ul) at 37°C or RNase A (20 μg/ml) for 30 minutes at 37°C and hybridized with FAM or HEX labeled oligo dT (Eurofins, custom oligos). Briefly, cells were washed twice with TM buffer (50 mM TrisHCl, pH 7.5, 3 mM MgCl2). Following the washes, the cells were incubated on ice with mild detergent (TM buffer with 0.4% Triton X-100, 0.2 mM PMSF, 0.5 mM CuCl2 and protease inhibitor) for 10 minutes. After washing with 1X PBS, the cells were treated with DNase 1 or RNase A. This was followed by washing and fixing the cells with 4% paraformaldehyde (PFA) for 10 minutes and subsequently with 100% cold methanol. After 10 minutes, the methanol was aspirated and 70% ethanol was added for 10 minutes to rehydrate the fixed sample. The ethanol was removed and cells were washed with 1 M Tris (pH 8) for 5 minutes. The cells were then incubated with the probe (5ʹ-labeled FAM oligo-dT or Hex oligo-dT, at a final concentration of 1ng/μl) in the hybridization buffer (Yeast tRNA-1 mg/mL, BSA-0.005%, Dextran sulfate-10%, Formamide, deionized-25% in 2X SSC) in a humidified chamber at 37°C overnight. After hybridization, the samples were washed with 4X SSC and 2X SSC sequentially, followed by mounting the samples using mounting media containing DAPI. Purified nuclei were treated with DNase 1/RNAse A as previously described and fixed with 4% PFA, following which they were treated similarly as was done for cells (see above).

### Chromatin fractionation, MNase treatment and Western blotting

Nuclei were isolated as previously described and 50ul was stored as the nuclear fraction. The purified nuclei were then suspended in chromatin elution buffer (10 mM Tris pH 7.4, 2 mM MgCl_2_, 2 mM EGTA, 0.1% Triton X-100) and increasing concentrations of NaCl at sequential incubations starting with 70 mM NaCl incubation at 4°C for 20 minutes. After 20 minutes, the nuclei were centrifuged at 400 g to release the weekly chromatin-bound proteins in the supernatant. The pellet was resuspended in the chromatin elution buffer with a higher concentration of NaCl (140 mM) and serially up to 590 mM of NaCl to collect protein fractions with varying strength of chromatin binding. Whole-cell, nuclear lysates and differential salt fraction supernatants were ran on 10% or 12% PAGE gels and transferred onto a nitrocellulose membrane. The membrane was blocked with 5% BSA-TBST [50 mM Tris (pH7.5), 150 mM NaCl, 0.1% Tween 20] for 1 hour at room temperature. Immuno-detection of the proteins was performed using the following antibodies Anti-Phosphoepitope SR proteins Antibody, clone 1H4 (Mouse Monoclonal Antibody) (MABE50, Sigma-Aldrich), Anti-SR protein family Antibody, clone 16H3 (MABE126, Sigma-Aldrich) and anti-SRRM1 protein (ab221061.To check for purity of the fractions the blots were probed for Tubulin (AC008, Abclonal), Stat3 (9139, CST), HDAC1 (5356, CST), HnRNP1A (sc32301, SantaCruz), and Histone H3 (2348, Abclonal) as these proteins show enrichment in different fractions.For MNase treatment the purified nuclei were incubated with MNase (M0247S, NEB) 1 U at 37°C for 30 minutes in a Ca^2+^ containing buffer (10 mM Tris pH 7.4, 2 mM MgCl_2_, 0.1 mM PMSF, 5 mM CaCl_2_). Post treatment, the supernatant was collected and used for DNA purification and Western blotting. Differential salt elution was also performed on MNase treated nuclear pellet as previously described.

## Results and discussion

### Nuclear Speckle (NS) morphology changes following genotoxic stress

**Figure 1.**
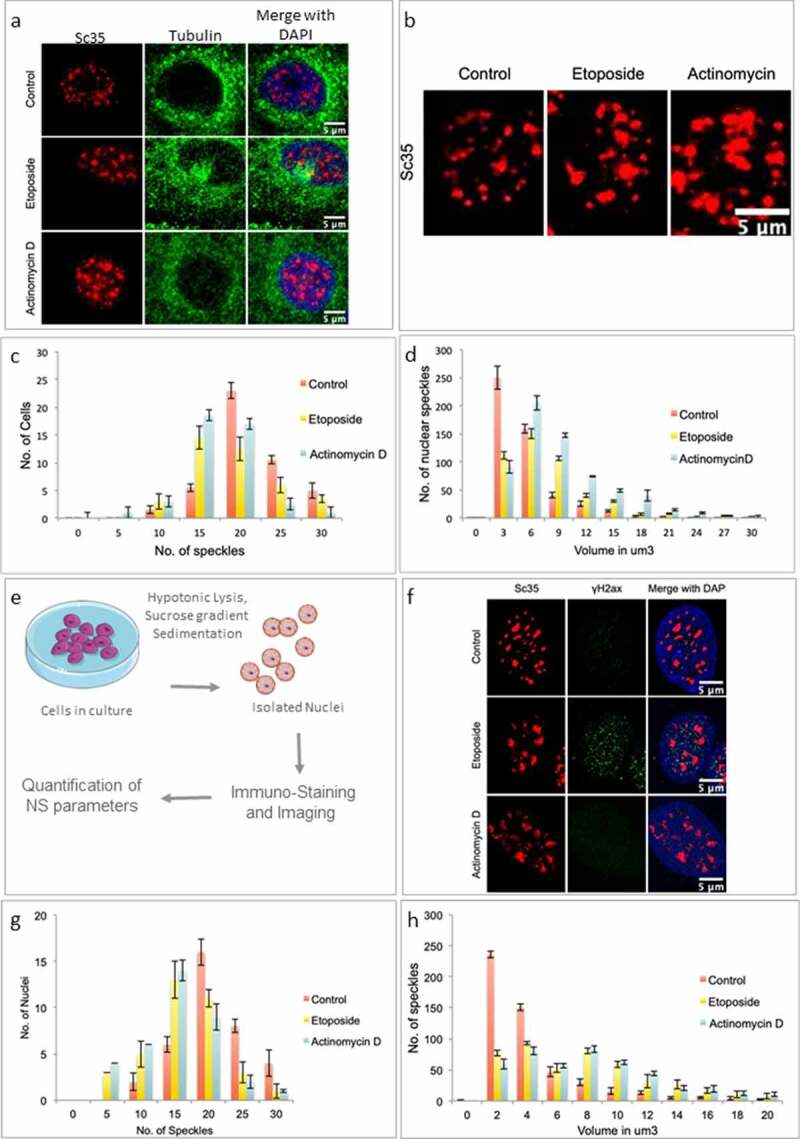
Nuclear Speckles exhibit changes in morphology upon stress. (a) Immunofluorescence for speckle marker Sc35 and Tubulin indicating changes in NS upon Etoposide and Actinomycin D treatment in fixed cells. (b) Representative confocal images for Sc35 Immunofluorescence under Control, Actinomycin D and Etoposide treatment. (c) IF analysis of NS number in maximum-intensity Z projections of Sc35 immunostaining after Control, Etoposide and Actinomycin D treatment. Quantification is depicted as the frequency distribution of NS number/cell. N = 3, n = 40. Error bars represent SD. (d) Frequency distribution of NS volume as measured by volumetric analysis of maximum-intensity Z projections of Sc35 immunostaining. N = 3, n = 40. Error bars represent SD. (e) Schematic describing purification of nuclei for immunostaining. (f) Representative images for immunostaining of Sc35 and γH2aX for purified nuclei upon Etoposide and Actinomycin D treatment. (g) Frequency distribution of Nuclear speckle number per nuclei as measured by the fluorescence intensity of Sc35. N = 3, n = 40.Error bars represent SD. (h) Frequency distribution of NS volume measured by volumetric analysis of Sc35 staining of purified nuclei. N = 3, n = 40. Error bars represent SD.

In the current study, we set out to explore the characteristics of NS changes following sublethal doses of double-strand breaks (DSBs) induced by Etoposide treatment in mammalian cells. As a comparison, we also performed Actinomycin D-mediated transcriptional inhibition as another case of genotoxic stress treatment. Transcriptional inhibition and osmotic stress have already been reported to change the NS morphology in several previous studies [15,27,28]. It was reported that NS became larger, rounder, and their number decreased. This was attributed to splicing factors diffusing back into speckles from active transcription sites and then accumulating within the speckle [29,30]. Recent studies supported this model by also proposing that long range motion and subsequent fusion of NS led to the larger NS with decrease in their number per nucleus [15]. In our study, Hek293 cells were either treated with DMSO (control) or Etoposide (10 uM, 4 hours) or Actinomycin D (40 uM, 4 hours), fixed and stained for Sc35, a prototypical NS marker and imaged using confocal microscopy (Figure 1(a,b)). Analyses of NS images across a large number of nuclei showed that NS morphology appeared to enlarge following both genotoxic stress treatment (DNA damage and Actinomycin D treatment) conditions. DNA damage by Etoposide was assessed by staining for γH2ax marker (a classical DDR marker), which revealed that the majority of cells were inflicted with DSBs as evidenced by their being positive to the DNA damage marker revealing discrete foci (Sup 1a& and). Concomitantly, quantitative analyses showed that the number of NS per cell decreased, accompanied, in parallel, by an increase in their average NS particle volume following DDR and transcriptional inhibition (Figure 1(a,d)). This has been represented as frequency distribution of number of nuclei versus the number NS and as number of speckles versus volume per speckle. To test for the significance of this frequency distribution change, we quantified the number of nuclei exhibiting less than 15 speckles. Such an analysis revealed a clear and significant increase in the number of nuclei with lesser NS (Sup 1 c). Similarly, we found significant decrease in the number of speckles with volume less than 3 um^3^ (Sup 1d). Such an analysis revealed that genotoxic treatment led to statically significant drop in the number of NS and increase in the speckle particle volume per nucleus in the population of cells as well as purified nuclei. Interestingly, these changes were observed to be reversible as reflected by the return of NS number and volume to normal distribution following 6 hours of cellular recovery (Sup 1a and 1b) suggesting that NS exhibit reversibly dynamic changes in morphology as well as the particle number following genotoxic stress. It appears that any stress in the genome leads to changes in NS organization leading to their particle coalescence into larger particles thereby leading to a drop in the average number of particles per cell as pointed out by previous studies as well.

Such chromatin-level changes leading to dynamic alterations in NS prompted us to address whether the NS show any direct association with chromatin. This query was also prompted by the recent findings in the field that NS serve as regulatory hubs for inducible gene expression changes that appear highly tunable to cellular needs [11]. Motivated by this elegant study, we wanted to test the level of NS association with nuclear chromatin in the purified mammalian nuclear system by probing for NS-chromatin association biochemically. We surmised that purified nuclei were experimentally much more amenable for biochemical probing and chromatin perturbation studies than whole cells. The experiments described below bare out our supposition. Using standard conditions, we performed mild hypotonic lysis of cells, released the nuclei and purified the same using sucrose density gradient centrifugation. Before proceeding to interrogate the NS in purified nuclei, we ascertained that NS morphological changes observed during genotoxic stress treatments (Etoposide or Actinomycin D treatments, as described above) performed on whole cells are also mirrored in isolated nuclear preparation.

### NS morphological changes associated with genotoxic stress are also observed in purified nuclei

The purified nuclei were washed, fixed, stained for Sc35/γH2ax and imaged (Figure 1(e)). As expected, the purified nuclei showed the nuclear bodies/ chromatin integrity and NS morphology very similar to that of whole cells. No discernible differences were observed in NS morphology in isolated nuclei *versus* the same in whole cells. Moreover, the NS dynamic changes observed during genotoxic stress treatments (Etoposide and Actinomycin D treatment) in whole-cell imaging were well recapitulated in the images retrieved with purified nuclei as well (Compare Figure 1(a,b) with Figure 1(f)). Quantitative analyses of a population of purified nuclei images revealed that the average number and volumes of NS matched well with that observed earlier in whole-cell preparation (compare Figure 1(c,d) with Figure 1(g,h), respectively). As observed earlier (Figure 1(a,d)), both genotoxic stress treatments resulted in cells where average NS particle size increased concomitant with the drop in their number even in purified nuclear preparation (Figure 1(f-h)).

It is has been proposed that certain active gene regions show a strong association with NS via nsaRNA (nuclear speckle-associated RNA) interacting with topological associated domains (TADs) [25]. However, the notion of NS association with chromatin has not received much attention so far as these particles were traditionally categorized for long as ‘inter-chromatin granule clusters’. This nomenclature historically well associated with NS implied that the NS particles were relatively free of chromatin anchorage and insoluble nuclear organelles [20,21]. However, more recent findings hint toward NS as phase-separated condensates that might get stabilized by nucleic acid association, suggesting that NS organization could be dynamic and the maintenance could depend on more complex extraneous interactions such as chromatin [25,31]. We, therefore, conjectured that ‘NS-chromatin’ association as a ‘biochemical possibility’ whose regulation might be tied up with their reported functions related to mRNA splicing [32,33]. We also envisaged that ‘NS-chromatin’ offers a plausible model for loci-specific splicing regulation in line with dynamic changes in chromatin.

### Test of ‘NS-chromatin’ association

#### Limited DNase 1 nicking leads to a collapse of NS-morphology

**Figure 2.**
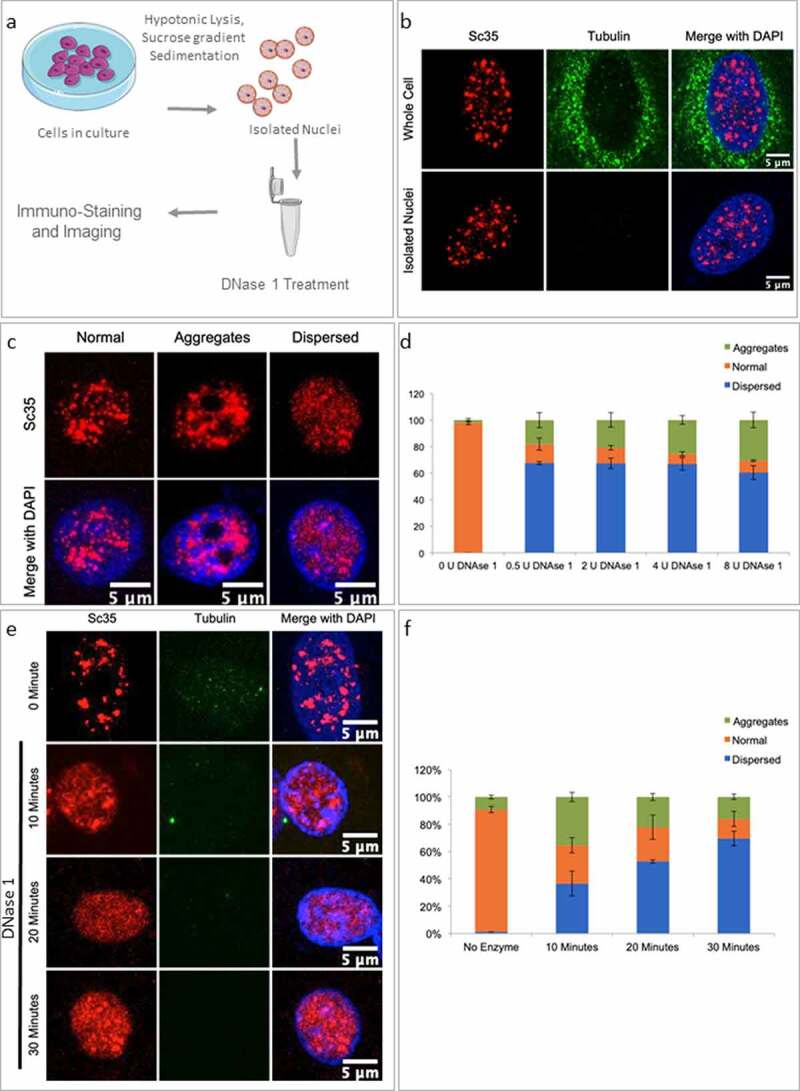
DNase 1 nicking leads to the collapse of NS-morphology. (a) Schematic for isolation of nuclei and DNase 1 treatment. (b) IF images for Tubulin (green) and Sc35 (red) staining in whole cells and purified nuclei. (c) Representative images of NS morphologies (Sc35 staining) observed upon DNase 1 treatment. (d) Quantification of different NS morphologies observed upon increasing concentrations of DNase 1. Error bars represent SD. (e) Representative images of Sc35 Immunostaining of Nuclei treated with DNase 1(2 U) over 10, 20, and 30 minutes of incubation. (f) Quantification of different NS morphologies observed upon DNase 1 treatment time course. Error bars represent SD.

In order to test the ‘NS-chromatin’ association hypothesis, we designed a minimally disruptive biochemical strategy (Figure 2(a)): We asked if nuclear chromatin were randomly nicked with a low level of (RNase-free) DNase 1 treatment in purified nuclei, one might observe significant morphological changes in NS resulting from the plausible ‘untethering effects’ of NS-particles from chromatin. Limited DNase 1 nicking in isolated mammalian nuclei has been used as a classical probing method for decades for interrogating transcriptionally active chromatin regions without incurring any large-scale untoward genomic alterations [34,35]. It is relevant to spell out the rationale behind this experiment clearly here: we surmised that if the native morphological characteristics of NS were critically dependent on their stable association with nuclear chromatin, limited DNase 1 nicking in nuclei would ‘untethered NS’, especially from the genomic loci that are transcriptionally poised or active. If, ‘NS-chromatin’ should act as an organizing scaffold for NS morphology and functions, as we hypothesize here, ‘untethered NS’ should disrupt NS morphology. In this analysis, following DNase 1 treatment of purified nuclei, the enzyme was inactivated by adding EDTA, the nuclei were pelleted and the supernatant was analyzed for released DNA. The nuclei were washed, fixed and stained for Sc35 to assess for changes in NS morphology if any. These treatments, by themselves without any DNase 1 addition, do not induce any alterations in NS morphology and we recovered images very similar to those obtained with whole-cell preparation (Figure 2(b)). In these conditions, nuclei treated with limited DNase 1 digestion revealed that DNase 1 nicked nuclei showed highly disintegrated NS with a nearly isotropic distribution of NS proteins in the nuclei as well as disorganized aggregates of NS proteins (Figure 2(c)). As argued earlier, typically, limited DNase 1 nicking is supposed to access ‘open’ regions of chromatin leading to no large-scale perturbation of the nuclear organization. Nuclear imaging (Sup 1i) and the recovery of a marginal amount of solubilized DNA (Sup 1g) revealed that the chromatin integrity was largely intact following such limited DNase 1 nicking of the purified nuclei. DAPI images of the nuclei showed that DNase 1 nicking had only resulted in some loss of DAPI-positive DNA where the nuclear morphology, integrity was well maintained (Sup 1i).

Interestingly, population analyses of NS images revealed that the majority of DNase 1 treated nuclei showed significant changes in NS morphology: as mentioned above, they were either dispersed all through the nucleus or showed NS particle aggregation phenotypes (Figure 2(c)). We quantified the percentage of nuclei exhibiting these changes as a function of varying concentrations of DNase 1 treatment. Quantification revealed that over 60% of the nuclei had dispersed NS and the NS aggregates also increased following DNase 1 treatment (Figure 2(d)). Increasing the duration of enzyme treatment also led to a time-dependent increase in the severity of NS dispersion and aggregation (Figure 2(e,f)). Even though it appears that NS aggregation precedes their dispersion in DNase 1 to isotropic protein distribution following treatment as reflected in the population analyses, it is unclear whether the two states are mechanistically related. Independent of whether aggregates precede NS dispersion or *vice-versa*, limited DNase 1 nicking clearly resulted in large-scale disruption of NS particle phase-separation in the nuclei (Figure 2(c-f)).

We reiterate here that the supernatant of the treated nuclei revealed that nuclease treatment did indeed result in a small fraction of nuclear DNA getting solubilized while most of the genome was largely intact (Sup 1g). We, therefore, believe that the conditions chosen for DNase 1 treatment were mild enough to induce limited nicking in the genome. The role of poly A RNAs and ncRNAs has been well documented in the maintenance of NS integrity [36], so we used DNase 1 reaction conditions on purified RNA to ensure there was no RNA degrading activity associated with DNase 1 treatment (Sup 1h). We, therefore, infer that DNase 1 treatment results suggest a possible relationship between NS anchorage on genomic chromatin whose disruption by inducing nicks in the genome lead to marked changes in the morphology of NS particles. We corroborated these findings further by the following set of experiments described below.

#### Limited DNase 1 nicking of isolated nuclei does not lead to any discernible changes in other nuclear substructures

In order to ascertain that the limited DNAse 1 nicking condition employed in our experiments did not lead to any discernible changes in the nuclear substructures, we probed for Nucleolar morphology using Nucleolin and NPM (B23) as markers, inner nuclear membrane using Emerin marker and the nucleoplasm marker distribution profile using HNRNPA1 protein marker.

**Figure 3.**
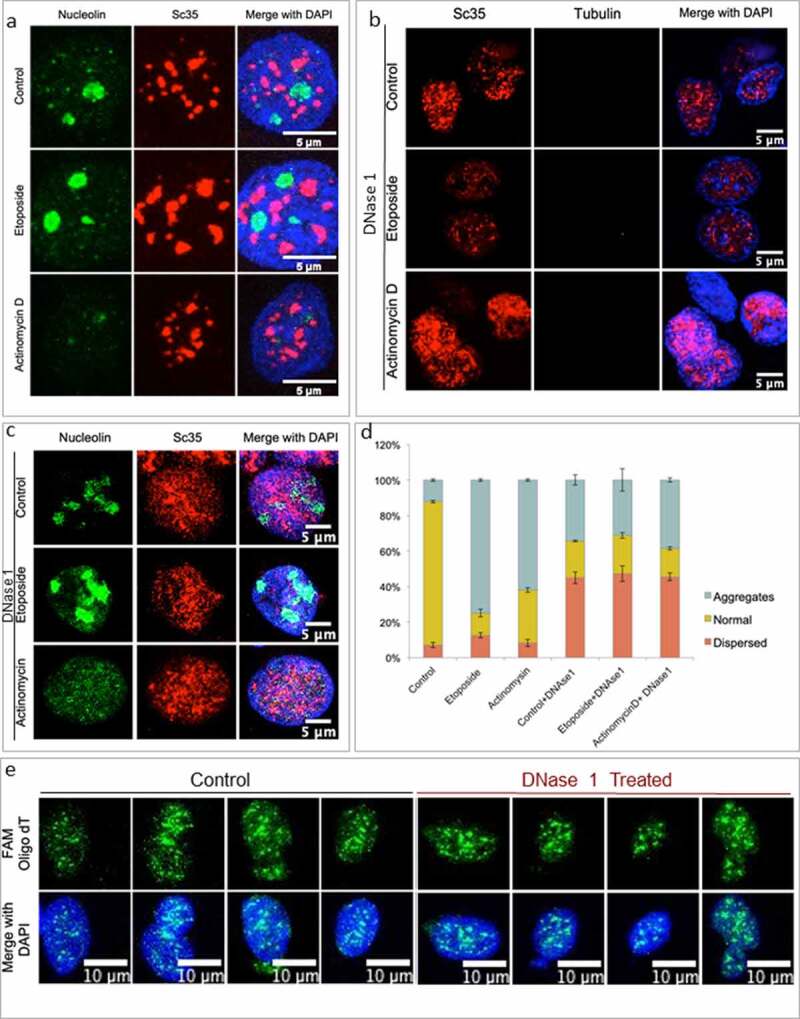
NS changes upon DNase 1 treatment are consistent over different physiological states. (a) Representative images for nuclei purified post Treatment of cells with Etoposide and Actinomycin D, stained for Sc35 and Nucleolar marker Nucleolin. (b) Representative IF images for nuclei purified after Etoposide and Actinomycin D treatment and subsequent DNase 1 (2 U) treatment. The Red channel marks NS staining and the green channel shows an absence of any Tubulin staining. (c) Nucleolin(green) and Sc35 (red) staining in DNase 1(2 U) treated nuclei post Etoposide and Actinomycin D treatment. (d) Percentage of NS morphologies observed post-DNase 1 treatment of purified nuclei from Etoposide and Actinomycin D treated cells. Error bars represent Standard deviation, N = 3, n = 50. e) Poly A mRNA FISH using FAM oligo dT for control and DNase 1 treated nuclei.

Nucleoli are highly dynamic and responsive phase-separated condensates, exhibiting stress-dependent changes in their morphology [37]. The DNase 1 treatment would nick the ‘accessible chromatin’ in the genome whereas Nucleolar organizer regions (NOR), being surrounded by heterochromatin [38], are unlikely targets of mild DNase 1 treatment, and therefore might not disrupt nucleolar integrity. DNase 1 treated nuclei exhibited nucleolar morphology very similar to that of control nuclei, which were not treated with the nuclease (compare control panels in Figure 3(a-c)). In the same conditions, a known disruptor of Nucleoli, namely Actinomycin D treatment, led to complete loss of Nucleolar morphology in both control and DNase 1 treated nuclei (Compare Actinomycin D panels in Figure 3(a-c)). Interestingly, Etoposide treatment (unlike Actinomycin D) does not lead to changes in Nucleolar morphology for both control and DNase 1 treated nuclei (Compare Etoposide lanes in Figure 3(a-c)), suggesting that purified nuclei provide a ‘robust’ system to capture nuclear substructure changes without incurring any untoward experimental artifacts. This conclusion was reinforced by the observation that while Nucleolar morphology was relatively refractory to limited DNase 1 treatment, the same nuclei exhibited marked disruption of NS morphology specifically in nuclease treated conditions (compare all panels of Sc35 staining in Figure 3(a) with respective panels in Figure 3(c)). Population analyses of purified nuclei corroborated the changes in NS morphology following DNase 1 nicking whose quantitative data is reflective of the trend observed in the earlier experiment (compare Figure 3(b-d) with Figure 2(c-f)).

Interestingly, inner nuclear membrane morphology was completely intact in DNase 1 treated nuclei, which was indistinguishable from that of control untreated nuclei (Sup. 1j). HNRNP nucleoplasm distribution, marked by HNRNPA1 protein staining was also unaltered by DNase 1 treatment (Sup. 1j). We also tested for NS behavior upon DNase 1 treatment using SON and SRRM1 proteins as additional NS markers, both of which also exhibited dispersal and aggregation upon DNase 1 treatment to varying extent, which was somewhat different from Sc35 staining. Such differences of NS specific markers could arise from their intrinsic differences in protein association with NS versus chromatin. (Sup. 2a-f). All these observations, put together, suggest that limited DNase 1 treatment of purified nuclei showed selective disruption of NS morphology while the overall distribution of other nuclear substructures, namely nuclear membrane, nucleoli, chromatin and nucleoplasm were relatively intact. Poly A mRNAs are also known to regulate NS integrity [36,39,40]. In order to test whether poly A mRNA distribution is affected by DNAse 1 treatment conditions, we performed Fluorescence in situ hybridization (FISH) for poly A mRNA using fluorescently labeled oligo dT probes.We observed that DNase 1 treatment did not seem to alter the poly A mRNA distribution in the nuclei (Figure 3(e,f)). The pattern of FISH signal were indistinguishable in the images of DNase 1 treated samples from that of untreated control samples.Expectedly, RNA FISH signals were largely eliminated following RNase A treatment (Sup 2 h). We believe that these results imply that the limited DNase 1 nicking specifically resulting in large-scale disruption of NS morphology is a strong indication that NS morphology is critically dependent on chromatin integrity, which is consistent with the NS-chromatin association model which we corroborated further in the experiments described below.

## NS proteins are tightly associated with chromatin: salt extraction and MNase probing experiments

### NS proteins are extracted from chromatin only at a high salt concentration

Histones and other tightly chromatin-bound proteins are extractable from chromatin scaffold only with high salt treatment [41]. This property has been well exploited for probing chromatin association of various proteins in the past [42]. In this model, nucleoplasmic proteins, being freely soluble in the nucleosol and therefore not bound to any scaffold or chromatin are recovered in the supernatant at low to physiological salt concentration (0.08 M-0.1 M NaCl), whereas those that are loosely bound to chromatin such as non-histone chromosomal proteins, transcription factors and other DNA repair/replication proteins etc are released at a modest salt concentration (0.1 M-0.3 M NaCl) [26,43]. In contrast, proteins that are relatively more tightly associated with chromatin get eluted out into the soluble phase and are recovered in the supernatant fraction only following high salt wash (above 0.3 M NaCl) and core histones which are the intrinsic components of chromatin are fully released from chromatin only at a very high salt concentration (1 M-2 M NaCl) [44]. Using this classical property of the high salt extractable nature of chromatin-bound proteins, we wanted to test whether NS component ‘SR proteins’ are also chromatin-associated. The purified nuclear system that we probed for DNase 1 nicking experiments described above came in handy for testing this property.

**Figure 4.**
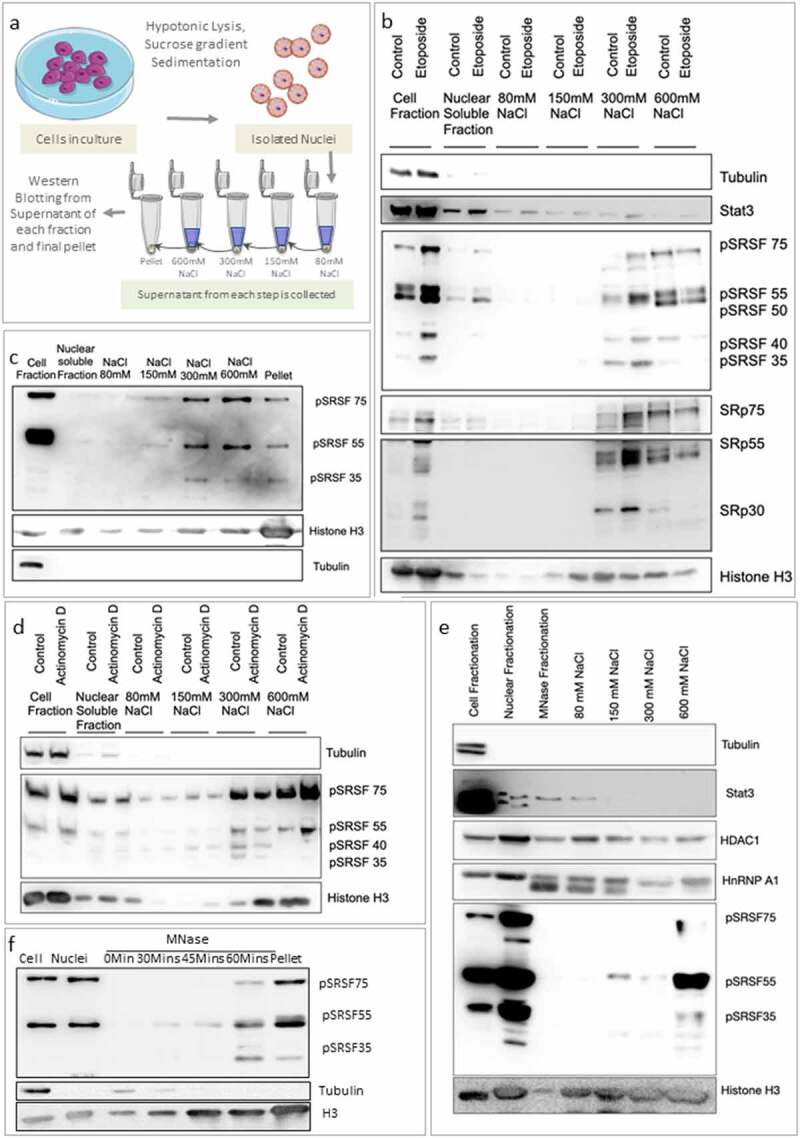
SR proteins exhibit strong chromatin association. (a) Schematic of Nuclear fractionation, sequential salt elution and Western blotting. (b) Western blots for supernatants released from chromatin after sequential salt elution. Consecutive lanes represent Control and Etoposide (10 uM, 4 hours) treated samples for each fraction. SR proteins (probed as pSRSF or SRSF) elute at higher salt concentrations compared to transcription factor Stat3. Histone H3 shows a stronger binding with maximum elution over 300 mM NaCl. Tubulin is used for indicating the purity of nuclear fractions. (c) Western blot for salt-based elution of the supernatants released and the ultimate chromatin pellet probed for SR proteins, Histone H3 and Tubulin. The blot shows clear enrichment of Histone H3 in the pellet and maximum elution of SR proteins at 600 mM NaCl. (d) Actinomycin D treatment (5 μg/ml) shows no alteration in chromatin association of SR proteins compared to DMSO control, as indicated by pSRSF Western blots. (e) Western blots for pSRSF proteins post MNase treatment followed by differential salt elution, probed for transcription factor Stat 3, chromatin remodeler HDAC1, and splicing factor hnRNPA1 showing weak chromatin association and H3 and SR proteins showing stronger association. (f) Western blot of supernatant released after MNase treatment of 30, 45, and 60 minutes and the remaining pellet.

We subjected purified nuclei to a regime of successively increasing salt washes as shown in the schematic (Figure 4(a)). Nuclei that were not treated with any salt served as a control to reveal intrinsically nuclear soluble proteins fraction (nucleoplasm fraction). Following successively higher salt treatment, we recovered supernatant fractions whose proteins were analyzed for Stat3/HDAC1 (transcription factors), NS proteins (SRSF proteins and their phospho versions) and Histones (H3 protein). This experiment revealed that while a large fraction of Stat3 protein was Nuclear soluble (no salt treatment), only a minor fraction of NS and histone proteins were recovered in the soluble pool. Interestingly, NS proteins began eluting out only at 0.3 M NaCl salt concentrations where further elution required as high as 0.6 M NaCl and above (Figure 4(b,c)). As expected core histone H3 reflected a similar high salt elution property. A discernible fraction of NS proteins were still associated with chromatin in the pellet fraction even after 0.6 M NaCl extraction (Figure 4(c)). Tightly bound core histone showed similar behavior where the majority of H3 was still associated with chromatin pellet even after 0.6 M NaCl extraction (Figure 4(c)). Interestingly, NS proteins, whether they were from the bulk fraction or the phosphorylated version, both exhibited similar high salt extraction properties where the proteins were eluted into the supernatant only at a salt concentration higher than 0.3 M NaCl. As the SR proteins are involved in steady state transcription, it is conceivable that the association of SR proteins as observed following salt elution is a function of transcription. To test this notion, we purified nuclei from Actinomycin D treated cells and observed that the chromatin association of SR proteins remained unaltered even after the cells were treated with genotoxic stress by Etoposide or by transcriptional dampening by Actinomycin D. (Figure 4(b-d)). This also raises the possibility that the association might be largely independent of of transcription. We also observed that the high salt elution property of NS protein remained unaltered whether the chromatin was prior-treated with MNase enzyme or not before performing salt extraction experiment (compare Figure 4(b-e)) suggesting that NS proteins exhibit stable chromatin association even in MNase digested chromatin. We also tested to see chromatin association of other NS proteins, SRRM1 and SON and found that both proteins associate strongly with chromatin, showing elution at 300 and 600 mM of NaCl (Sup 2i). This result strongly suggested that NS proteins were indeed tightly associated with chromatin.

### MNase released soluble mono-dinucleosomes also leads to a concomitant release of NS proteins from genomic chromatin

One of the simplest biochemical tests of any chromatin-associated factor is its ability to corelease with the soluble fraction following MNase digestion of total nuclear chromatin. We performed this test on isolated nuclei incubated in MNase compatible buffer followed by initiating MNase digestion reaction. MNase is an endonuclease that digests chromatin selectively at the linker regions thereby releasing oligonucleosomes in the soluble chromatin fraction, where an increasing time of enzyme digestion leads to a relatively higher level of mono-dinucleosomes released in the soluble chromatin fraction recovered in the supernatant [45]. MNase digestion time course revealed that a significant fraction of chromatin was released as soluble fraction consisting of mono-dinucleosomes from the genomic chromatin by about 60 minutes of MNase digestion (Sup. 1i). The soluble chromatin supernatant fractions were analyzed for SR proteins and H3 histone proteins where we observed that an increasing fraction of NS proteins were released into supernatant as a function of MNase digestion time course (Figure 4(f)). NS protein release during MNase digestion time course paralleled the release of Histone proteins, represented by H3. Interestingly, a measurable fraction of NS and Histone proteins were still retained in the undigested part of chromatin as the pellet fraction (Figure 4(f)).

**Figure 5.**
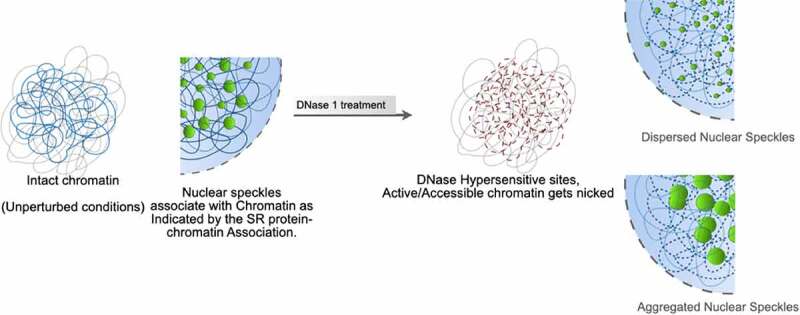
Model: In unperturbed conditions nuclear speckles (NS) and chromatin are closely interacting as indicated by the strong association of NS constituent SR proteins with chromatin. Such an interaction might act as a ‘tether’ for NS. Perturbations like limited DNase 1 nicking of chromatin leads to ‘untethering’ of NS from chromatin which results in destabilization of NS into isotropic distribution or aggregation of constituent proteins as observed by the immunostaining.

Taken together, all these results point toward an interesting new model (Figure 5) of NS organization that involves its close association with nuclear chromatin. This conclusion is drawn based on several important observations made in the current study: 1. Limited DNase 1 nicking of chromatin seems to ‘untether’ NS particles leading in turn to their disorganization as reflected by larger aggregates of NS in some nuclei and complete dissolution of the same in some other nuclei. 2. High salt elution of NS proteins reflective of their stable association with chromatin. 3. Corelease of NS with mono-dinucleosomal soluble chromatin following MNase digestion of nuclei.

## ‘NS-chromatin’ model: concluding thoughts

NS, conventionally and historically, were regarded as interchromatin granules, whose subsequent cell biological characterization unveiled that they were functionally very important intranuclear bodies. Several structural and functional studies related to NS strongly posit that their association with chromatin was very dynamic and supposedly transient [14,46] Chromatin itself being rather dynamic poses an interesting challenge on the NS relationship. It is in this context; our current study contributes to an important clarification of the system comprising of NS-chromatin interaction. We show that NS, notwithstanding their status of being referred to as interchromatin granules, are in fact very closely and strongly associated with chromatin. Microscopic studies tend to miss out on this aspect due to several technical reasons: at a general level, NS colocalization with chromatin fibers has not been easy to pin down as the bulk chromatin images preclude any close scrutiny of chromatin fibers that anchor NS; only in cases where specific gene loci were monitored, colocalization with NS was discernible in microscopy images [11,46]. Systematic biochemical studies that probe the nature of NS association with chromatin or any other nuclear scaffold structures have been largely missing in the field because of which neither the proteome-dynamics of NS nor its relationship vis-à-vis chromatin changes have been uncovered so far.

We believe that our current study is not only novel but also very significant in the field for the following reasons. The current study sets up a simple framework to start rethinking of NS as a novel biochemical entity that is ‘in-continuum’ with chromatin. We strongly believe that the evidence provided in the current study does justify this rethink. In fact, such a rethink does not contradict the existing models of NS as dynamic interchromatin granule structures. Our model (figure 5) only tends to extend the conventional dynamic interchromatin granule concept model into a more generally acceptable, chromatin-centric NS model where the particles occupy a closer, stably dynamic association with chromatin. In this new model, one would then have to wonder about the interacting partners between NS and chromatin that lead to such putative stable association. Several candidates might stand up to fit the bill, all of which need careful and detailed investigation as a part of a separate study.

The candidates that look attractive in mediating the association between NS and chromatin are possibly RNA (coding or noncoding) associated with chromatin, the histone proteins, even the post-translationally modified histones or some tightly bound adapter proteins. Recent studies have hinted toward possible ‘stable’ NS-chromatin interactions mediated via specialized chromatin structure G-quadruplexes (G4s)^31^. G4s are non-canonical four-stranded DNA structures comprising of repetitive guanines (G) forming stable stacked tetrad structures [47]. These structures have been implicated in gene regulation, replication, DNA repair and Telomere protection [48]. Komurková et al. reported that upon immunostaining G4s, highest degree of colocalization was observed with transcription factories marked by phosphorylated RNA pol II and NS marked with Sc35^31^. Interestingly, the G4s have been reported in highly active, GC rich euchromatin. The GC rich regions have been implicated for NS formation in earlier studies as well. Jabbari et al. reported that genomic sequence can dictate nuclear body formation, with high GC density positively correlating with NS [49]. The idea of critical NS-chromatin association is also supported by findings where knockdown of major NS component Srrm2 led to disruption of NS and a reduction in intra- and inter-TAD interactions. This hints toward a critical role of NS in genome organization[50]. Recently, nucleosomal proteins have also been reported to mediate NS-Chromatin interactions. Histone variant H2A.B.3 was identified to associate with multiple NS proteins including SF3B1, DDX42, and SNRNP70 in mice sperm. The same study also reported localization of H2A.B.3 at NS, further supporting a NS-chromatin association model [32]. Direct interaction of NS with chromatin is further strengthened by a recent ChIP-seq based study reporting that RNA binding proteins (RBPs) occupy various genomic loci, suggesting that various resident RBPs of NS can facilitate chromatin association as well [51]. In light of these supporting studies, our model lends novel insights into the field of nuclear organization of NS and chromatin and paves the way for detailed research of specific candidates that govern such nuclear body and DNA interactions.

It will be very interesting to systematically probe the association dynamics of NS *vis-à-vis* different states of chromatin (transcriptionally poised/bivalent/active/repressed, etc.) and establish the nature of communication between the changes in NS-proteome *versus* multiple chromatin functional states. We believe that this aspect of research has been the missing puzzle in the mystery of NS biochemistry.

## Supplementary Material

Supplemental Material
