# Supplementary material for: Mammalian nuclear speckles exhibit stable association with chromatin: a biochemical study
Source: Nucleus. 2022 Feb 27;13(1):58–73. doi: 10.1080/19491034.2021.2024948 (PMC8890396; doi:10.1080/19491034.2021.2024948)
Supplement: Supplemental Material [file KNCL_A_2024948_SM5157.zip › supplementary/s1.pdf]

Supplementary Figure :1

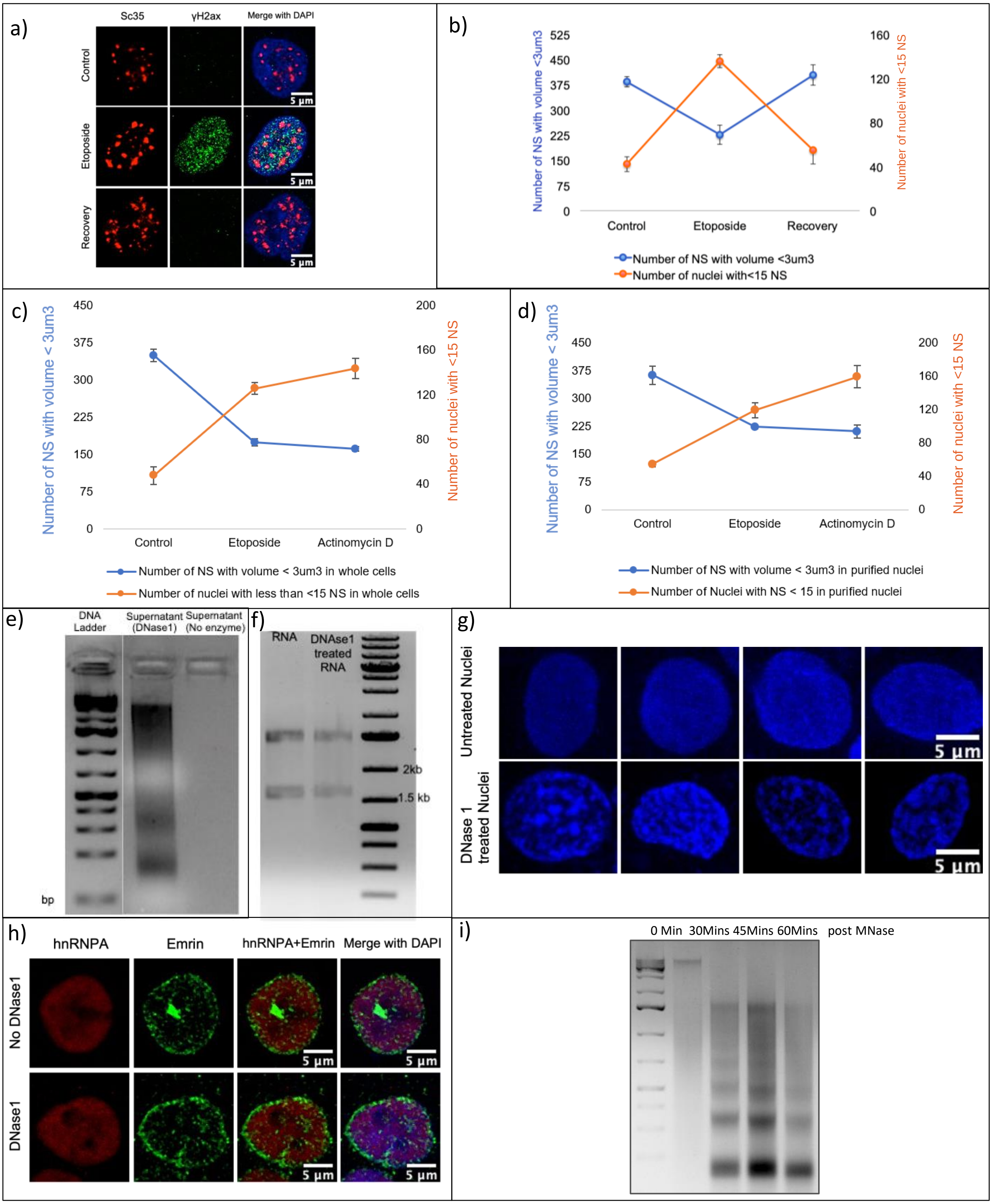

a) Representative images for NS and γH2ax upon Etoposide treatment and Recovery.
b) Quantification for the NS parameters upon Recovery showing reversible nature of NS behaviour.
c) Quantification of NS number and volume in whole cell. ($P < 0.05$) Unpaired t test
d)Quantification of NS number and volume in purified nuclei ($P < 0.05$) Unpaired t test.
e) Agarose gel for the Supernatant collected post DNase 1 treatment of purified nuclei showing digested DNA.
f) Purity of DNase1 showing intact RNA upon DNase Treatment.
g) DNA staining using DAPI post DNase1 Treatment.
h) Emerin and hnRNPA staining upon Control and DNase1treatment of purified nuclei showing intact nuclear lamina and Nuclear soluble proteins.
i)Agarose gel analysis of DNA extracted from nuclei during a time course of MNase treatment.
