# Supplementary material for: Mammalian nuclear speckles exhibit stable association with chromatin: a biochemical study
Source: Nucleus. 2022 Feb 27;13(1):58–73. doi: 10.1080/19491034.2021.2024948 (PMC8890396; doi:10.1080/19491034.2021.2024948)
Supplement: Supplemental Material [file KNCL_A_2024948_SM5157.zip › supplementary/s2.pdf]

Figure: Supplementary 2

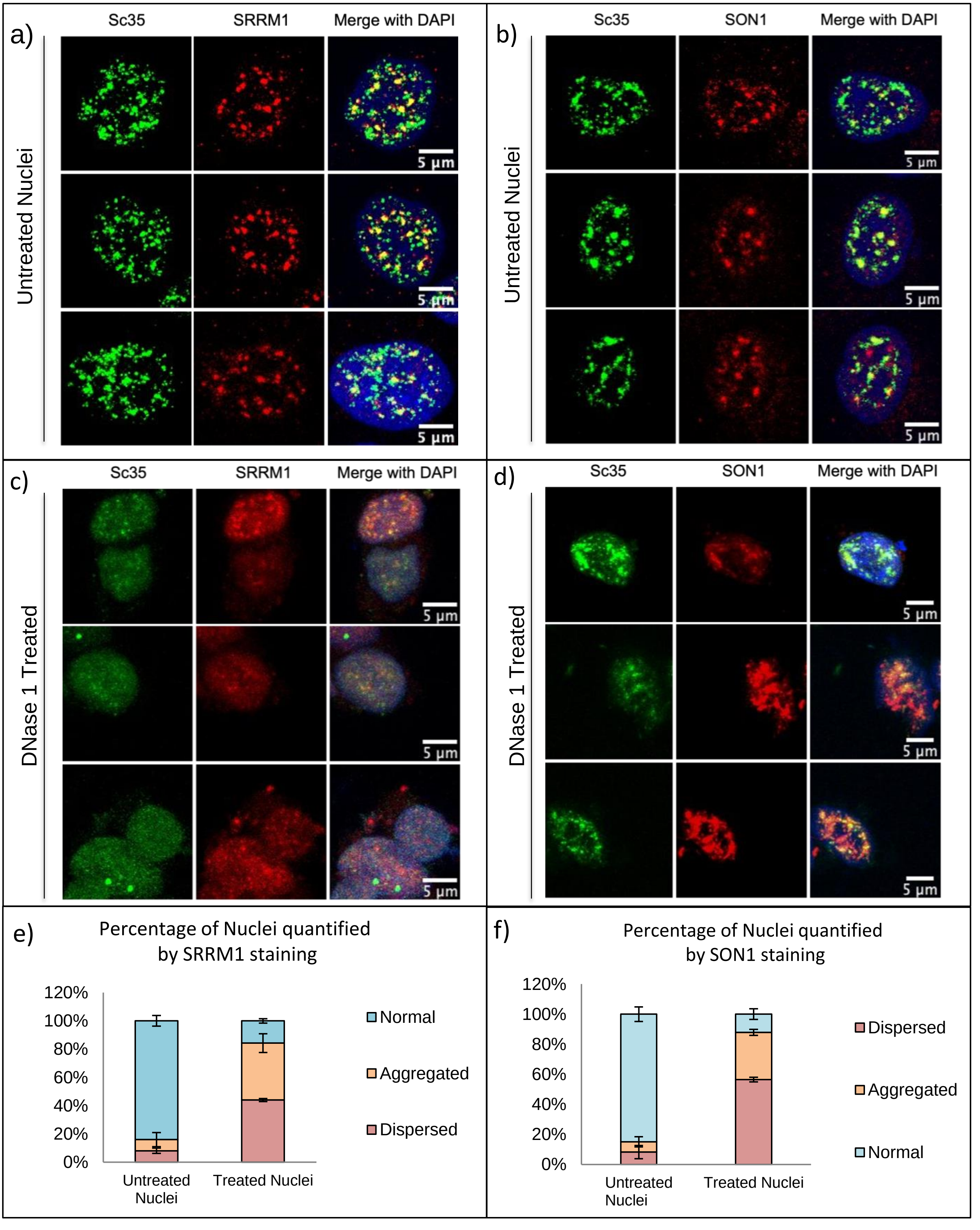

a) IF staining of Sc35 and SRRM1 in purified nuclei.
b) IF staining of Sc35 and SON 1 in purified nuclei .
c) IF staining of Sc35 and SRRM1 in purified nuclei post DNase 1 treatment.
d) IF staining of Sc35 and SON1 in purified nuclei post DNase 1 treatment.
e) Percentage of NS morphologies as stained by SRRM1 observed post-DNase 1 treatment of purified nuclei. Error bars represent Standard deviation, N=3,n=50
f) Percentage of NS morphologies as stained by SON1 observed post-DNase 1 treatment of purified nuclei. Error bars represent Standard deviation, N=3,n=50
