# Supplementary material for: Mammalian nuclear speckles exhibit stable association with chromatin: a biochemical study
Source: Nucleus. 2022 Feb 27;13(1):58–73. doi: 10.1080/19491034.2021.2024948 (PMC8890396; doi:10.1080/19491034.2021.2024948)
Supplement: Supplemental Material [file KNCL_A_2024948_SM5157.zip › supplementary/s3.pdf]

Figure: Supplementary 3

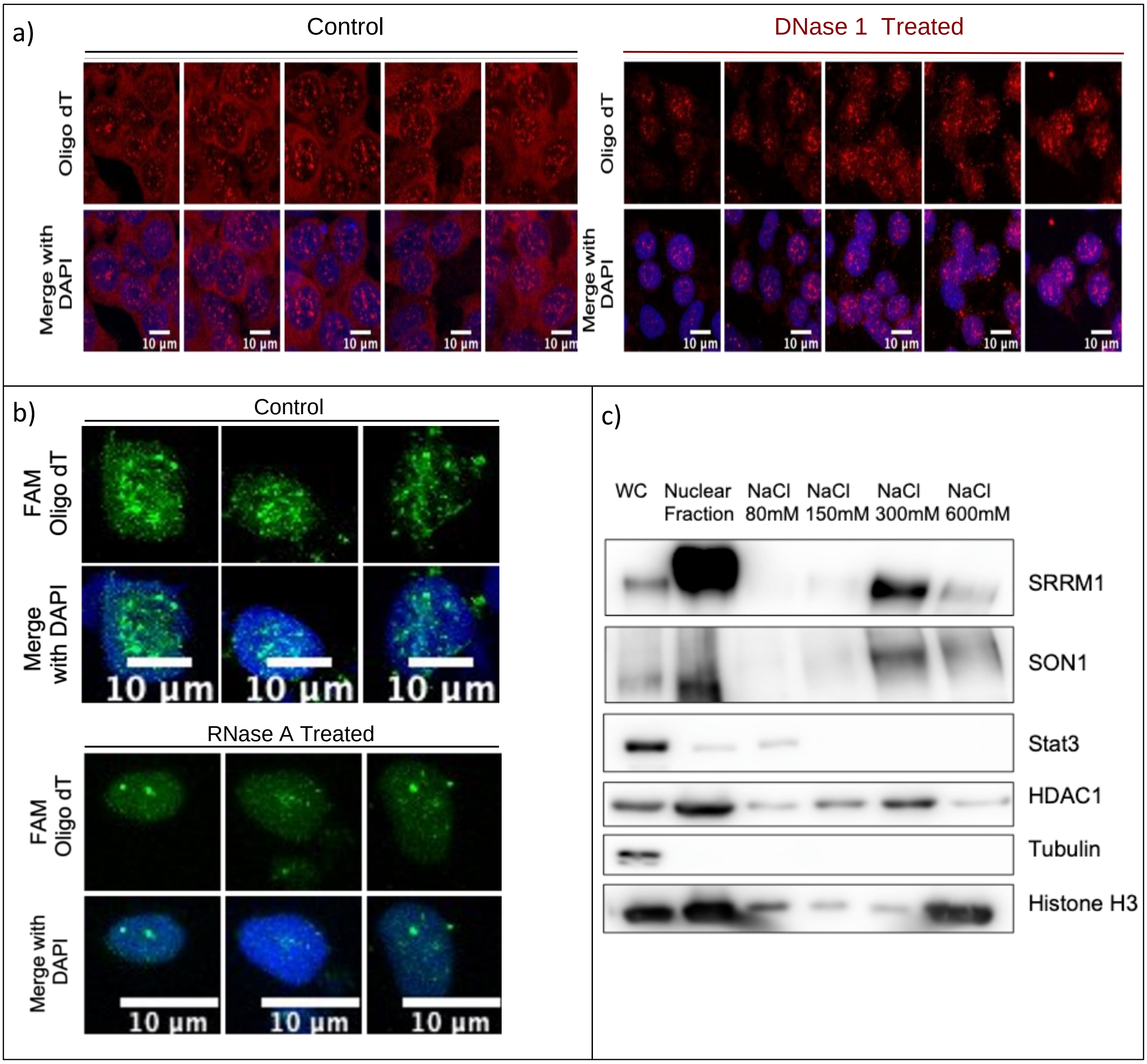

a) Poly A RNA FISH using FAM labeled oligo dT in control and DNase 1 treated cells.
b) Poly A RNA FISH in nuclei for control and RNase A treated cells.
c) Western blot for proteins eluted from chromatin post serial salt based elution. SRRM 1 and SON 1 also show elution at higher salt concentration like other SR proteins.
